# Genetic Encoding of a Non-Canonical Amino Acid for the Generation of Antibody-Drug Conjugates Through a Fast Bioorthogonal Reaction

**DOI:** 10.3791/58066

**Published:** 2018-09-14

**Authors:** Benjamí Oller-Salvia

**Affiliations:** ^1^Medical Research Council Laboratory of Molecular Biology

**Keywords:** Chemistry, Issue 139, Antibody-drug conjugates, bioorthogonal reactions, cyclopropene, drug delivery, protein engineering, bioconjugation

## Abstract

Antibody-drug conjugates (ADCs) used nowadays in clinical practice are mixtures of antibody molecules linked to a varying number of toxins at different positions. Preclinical studies have shown that the therapeutic index of these traditional ADCs can be improved by the site-specific linkage of toxins. However, current approaches to produce homogeneous ADCs have several limitations, such as low protein expression and slow reaction kinetics. In this protocol we describe how to set up an expression system to incorporate a cyclopropene derivative of lysine (CypK) into antibodies using genetic code expansion. This minimal bioorthogonal handle allows rapid conjugation of tetrazine derivatives through an inverse-demand Diels-Alder cycloaddition. The expression system here reported enables the facile production and purification of trastuzumab bearing CypK in each of the heavy chains. We explain how to link the antibody to the toxin monomethyl auristatin E and characterize the immunoconjugate by hydrophobic interaction chromatography and mass spectrometry. Finally, we describe assays to assess the stability in human serum of the dihydropyridazine linkage resulting from the conjugation and to test the selective cytotoxicity of the ADC for breast cancer cells with high levels of HER2 receptor.

**Figure:**
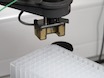


## Introduction

Antibody-drug conjugates (ADCs) combine the selectivity of biotherapeutics and the potency of small cytotoxic molecules. Most ADCs aim to decrease the side effects of traditional chemotherapy by targeting drugs that affect DNA or microtubule polymerization to cancer cells^1^. First-generation ADCs approved by the Food and Drug Administration (FDA) rely on the modification of lysines and cysteines, which generates mixtures of molecules modified at different positions with decreased pharmacokinetic properties^2^. By contrast, site-specific conjugation of drugs to the antibodies can generate compounds with improved therapeutic indeces^3^,^4^. Seeking to address the challenge of producing homogeneous ADCs, several selective chemical and enzymatic modifications have been reported^1^,^5^. However, current methods can target only certain position on the antibody, suffer from low protein expression, provide linkers with low stability, or rely on slow and low-yielding reactions.

Incorporation of non-canonical amino acids (ncAA) through genetic code expansion enables the site-specific installation of a plethora of bioorthogonal reactive groups into proteins, potentially overcoming the limitations of other methods used to generate ADCs. Encoding ncAAs in response to a target (stop) codon relies on aminoacyl-tRNA synthetase/tRNA pairs that are orthogonal to the endogenous pairs that incorporate canonical amino acids^6^. Several ncAAs have been incorporated into antibodies to generate ADCs. However, most suffer from various liabilities for applications in therapeutic drug conjugation. p-acetylphenylalanine (pAcF)^7^,^8^ is not fully bioorthogonal, requires low pH (4.5) and long reaction times (> 60 h), while azides such as p-azidophenylalanine (pAzF)^7^,^9^,^10^, p-azidomethylphenylalanine (pAMF)^11^, and an azide derivative of lysine (AzK)^12^,^13^ may be reduced in the cell^14^, and the copper used to catalyze Huisgen cycloadditions can induce oxidative damage^15^.

Although alternative ncAAs based on trans-cyclooctene (TCO), cyclooctyne (SCO) and bicyclo[6.1.0]nonene (BCN) have recently been encoded in an antibody for bio-imaging purposes, the expression system suffers from very low yields (0.5 mg/L)^16^. Moreover, cyclooctenes and cyclooctynes are large and hydrophobic handles that may increase the susceptibility of the ADC for aggregation -ADC payloads are traditionally hydrophobic and the physicochemical properties of the linker have been shown to greatly impact pharmacokinetics and therapeutic index^17^. By contrast, 1,3-disubstitued cyclopropenes are small reactive groups that should cause minimal alteration in the protein structure and physichochemical properties^18^. Cyclopropenes selectively and rapidly react with tetrazines via an inverse electron-demand Diels-Alder cycloaddition^19^. In this protocol we make use of a derivative of lysine (CypK, Figure 1b) bearing a methyl-cyclopropene that is less affected by steric hindrance than larger strained unsaturated cycles and has a reaction rate constant in the order of 1-30 M^-1^s^-1^ in aqueous media^18^,^20^.

We recently reported how to incorporate CypK into antibodies to generate ADCs by reacting this minimal bioorthogonal handle with tetrazine-bearing molecules^21^. Here we describe the ADC preparation procedure in more detail with emphasis on the most challenging steps. The incorporation of CypK is directed using a pyrrolysyl-tRNA synthetase(PylRS)/tRNA_CUA_(PylT) pair in response to an amber codon introduced in the antibody heavy chain (HC)^22^. Here we use two plasmids for transient transfection (Figure 1a), one encoding the heavy chain of the antibody and the other one encoding the light chain (LC), both containing the PylRS/PylT cassette. Alternatively, a stable cell line that enables higher antibody yields can be generated through a more laborious procedure^21^.

The aforementioned expression systems can produce the therapeutic anti-HER2 immunoglobulin 1 (IgG1) trastuzumab with CypK at similar levels to the wild type antibody. We selected the first position of the CH1 domain on the heavy chain to encode the ncAA (HC-118TAG). This is the most commonly modified site in ADCs^23^ and is known as HC-118 (EU numbering) but has also been referred to as HC-121 (sequence position) and HC-114 (Kabat numbering)^24^. Since this position is conserved throughout all IgG1s, these expression systems should be amenable to most therapeutic antibodies.

We show trastuzumab(CypK)_2_ can be easily purified by protein A followed by fast protein liquid chromatography with a hydrophobic interaction column (FPLC-HIC). Subsequently the antibody is covalently linked within 3 h to the microtubule polymerization inhibitor monomethyl auristatin E (MMAE), which is used in the FDA-approved ADC Adcetris. Here we use a benzyl-tetrazine derivative of MMAE (tetrazine-vcMMAE) with a linker comprising a glutarate spacer and a valine-citrulline protease-labile component followed by a *p*-aminobenzylalcohol self-immolative unit; this linker is cleaved by Cathepsin B in the lysosome upon internalization of the ADC resulting in the traceless release of the toxin^25^. In order to show the broad scope of the reaction, the antibody is also linked to the fluorophore tetramethylrhodamine (TAMRA). We explain how to verify the identity of the conjugate by liquid chromatography coupled to mass spectrometry (LC-MS) and to calculate the drug-to-antibody ratio (DAR) using high performance liquid chromatography with a hydrophobic interaction column (HPLC-HIC).

As part of the characterization of the antibody performance, we describe how to test the stability of the dihydropyridazine linkage in human serum. This parameter is more easily assessed in trastuzumab-TAMRA because it can be quantified by a simple ELISA and the interpretation of the results is not complicated by the protease labile component of trastuzumab(MMAE)_2_. Finally, the selectivity and potency of trastuzumab(MMAE)_2_ is assessed by comparing the cytoxicity of the ADC across cell lines expressing different levels of HER2. This assay also provides a functional proof of the ADC stability when performed after incubating the immunoconjugate in human serum.

## Protocol

### 1. Produce and Characterize the Antibody

1. Express the antibody
  1. Thaw a vial of HEK suspension cells in a 250 mL flask containing 50 mL of expression medium supplemented with 100 units/mL penicillin, 100 µg/mL streptomycin, and 250 ng/mL amphotericin B. Keep the cells at 37 °C with 8% CO_2_ in humidified incubators equipped with a shaker at 125 rpm. Split cells to 0.3-0.5 x 10^6^ cells/mL (every 2-3 days) at least 2 times before transfecting.
  2. When a density of 2.5 x 10^6^ cells/mL is reached (2-3 days after splitting), prepare a fresh solution of 100 mM CypK. For this purpose, weigh 64 mg of CypK, add 2.5 mL of 0.1 sodium hydroxide, vortex, spin down to recover all undissolved particles and sonicate.
  3. Add 2.5 mL of CypK (100 mM in 0.1 M NaOH) to 42.5 mL of expression medium supplemented with antibiotics. Mix well, add 250 µL of 0.1 M HCl, and sterilize using a 0.22 µm filter.
  4. Dilute 50 µg of HC and LC pKym1 plasmids^21^ to 2.5 mL with reduced serum medium. In a separate tube, dilute 135 µL of transfection reagent to 2.5 mL with reduced serum medium.
  5. Five minutes after preparing the solutions, mix the plasmids and the transfection reagent solution and incubate for 20 min to allow the formation of complexes between the DNA and the transfection reagent.
  6. In the meantime, centrifuge 125 million cells at the target density for 5 min at 500 x g, resuspend with the expression medium containing CypK and add the DNA–transfection reagent mixture. CypK can be purchased or synthesized as reported previously^18^.
  7. After incubating cells for 20 h, add 250 µL of transfection reagent enhancers included in the kit.
  8. Harvest antibodies from the supernatant 6-7 days after addition of CypK (no change of medium is required during expression). *Alternatively, to obtain higher and more consistent yields, a stable cell-line can be generated as described in Oller-Salvia* et al. *2018^21^. In this case, trastuzumab(CypK)_2_ is expressed simply by addition of CypK 5 mM in the expression medium.
2. Purify the antibody
  1. Centrifuge the cells for 15 min at 3000 x g.
  2. Filter the supernatant with a 0.45 or a 0.22 µm filter. If the filter becomes occluded, replace it for a new one and continue filtering. If this happens after only a few milliliters, centrifuge the supernatant for an additional 15 min at 7000 x g.
  3. Add protein A resin (2 mL/100 mL of supernatant) in an empty polypropylene chromatography column and equilibrate the resin with at least 5 volumes of bead wash buffer (0.1 M sodium phosphate, 150 mM NaCl).
  4. Split the supernatant into two 50 mL conical tubes and add 5x bead wash buffer (0.5 M sodium phosphate, 150 mM NaCl) followed by the pre-equilibrated protein A resin. Place the conical tube on a roller for 3 h at room temperature to pull down the antibody in the supernatant. Alternatively: Add the supernatant on the column with at least double the recommended volume of resin and allow to flow through. Make sure there is not a significant amount of antibody left in the supernatant by SDS-PAGE. If there is, elute the supernatant through the resin once again.
  5. Transfer protein A resin with the supernatant into a column and allow the liquid to flow through.
  6. Add 25 mL of wash buffer (or at least 10 resin volumes) and allow to flow through.
  7. Elute the antibody with 4 mL of 0.1 M sodium citrate, pH 3, on 1 mL of 1 M phosphate buffer, 150 mM NaCl.
  8. Dilute the antibody with 10 mL of PBS, concentrate to 0.5-1 mL by centrifugal filtration, and exchange buffer three times with PBS.
  9. Purify the samples by FPLC using a butyl HIC column with a flow of 0.5 mL/min and a 0-100% gradient in 30 min of low-salt buffer (50 mM sodium phosphate pH 7.0, 20% isopropanol) in high-salt buffer (1.5 M (NH4)_2_SO_4_, 50 mM sodium phosphate pH 7.0, 5% isopropanol). Collect all fractions and monitor the elution at 280 nm. Small amounts (< 1 mg) can be purified with HPLC-HIC under the conditions described in 2.4 to maximize yield and purity.
  10. Pool the fractions that contain antibody, dilute them to at least 4 mL with PBS, concentrate them by centrifugal filtration and exchange buffer three times with PBS.
3. Quantify the antibody
  1. Obtain an approximate concentration by measuring absorbance at 280 nm using a micro-volume spectrophotometer.
  2. If an accurate measurement is required, use an ELISA kit to measure human IgGs. For a rough estimate, use SDS-PAGE with a Coomassie-based stain as follows:
    1. Prepare standards by diluting six times two-fold a trastuzumab standard quantified by ELISA at 1 mg/mL.
    2. Boil the standards and the samples at approximately 0.25 mg/mL in reducing loading buffer.
    3. Run them in a 4-12% Bis-Tris SDS-PAGE using MES-SDS buffer and stain with a Coomassie-based dye. Finally measure color density of the band corresponding to the light or the heavy chain using ImageJ and interpolate the signal from the samples in the standard curve.
4. Store the antibody at 4 °C.

### 2. Conjugate the Antibody and Characterize the ADC

1. Conjugate the antibody with the tetrazine-bearing molecule
  1. Dilute 10 molar equivalents of tetrazine-vcMMAE (4 µL, 3.4 mM in dimethyl sulfoxide (DMSO)) with 20 µL of acetonitrile and 76 µL of PBS in a small conical tube (*e.g.,*PCR tube).
  2. Add 1 molar equivalent of trastuzumab(CypK)_2_ (100 µL, 2 mg/mL in PBS), mix thoroughly, and allow to react for 3 h at room temperature (25 °C) to form trastuzumab(MMAE)_2_. Note: Other molecules such as tetrazine-5-TAMRA can be linked to the antibody instead of tetrazine-vcMMAE using this protocol.
2. Purify the ADC
  1. Pre-equilibrate a size exclusion spin column with PBS following the manufacturer’s instructions.
  2. Add the whole volume of the reaction on the spin column and centrifuge at 1500 x g for 1 min.
3. Quantify the ADC and analyze the conjugate by SDS-PAGE
  1. Quantify the ADC as described in 1.3.2. by measuring the color density of the light chain on an SDS-PAGE using the non-modified antibody for the standard curve.
  2. The heavy chain should have slightly shifted showing an increase in molecular weight upon the conjugation. Note: If the antibody is modified with a fluorophore such as in trastuzumab(TAMRA)_2_, only the band corresponding to the heavy chain should show in-gel fluorescence before staining.
4. Analyze the conjugate by HPLC-HIC
  1. Equilibrate the HPLC-HIC column with 100% buffer A (1.5 M (NH_4_)_2_SO_4_, 50 mM sodium phosphate pH 7.0, 5% isopropanol) for 5 min.
  2. Mix 15 µL (67 pmol) of a 1 mg/mL solution of trastuzumab(CypK)2 with 15 µL of 2x buffer A in a vial. Then program a 10 µL injection in the HPLC.
  3. Elute in an isocratic 1 mL/min flow with 100% buffer A for 1 min followed by a 15 min gradient from 100 to 0% of buffer A in B (50 mM sodium phosphate pH 7.0, 20% isopropanol). Monitor the elution at 280 nm.
  4. Calculate the DAR by integrating the peak corresponding to each species and using the resulting areas in the following equation: DAR = (1x trastuzumab(MMAE) + 2x trastuzumab(MMAE)_2_) /(trastuzumab(MMAE)_0_ + trastuzumab(MMAE)_1_ + trastuzumab(MMAE)_2_) Expected retention time for trastuzumab(CypK)_2 _is 7.5-8.0 min, for trastuzumab(CypK, MMAE) is 9.1-9.6 min, and for trastuzumab(MMAE)_2_ is 10.5-11.0 min.
5. Analyze the conjugate by LC-MS
  1. Deglycosylate 30 µL of 1 mg/mL ADC and the unmodified antibody in non-reducing conditions using 250 PNGase F units for at least 6 h at 37 °C.
  2. Elute the antibody into the mass spectrometer in a C4 1-5 µm 1.0 x 100 mm column using a 20 min gradient of 2% to 80% acetonitrile in water. Acquire data over an m/z range of 350-4000 in positive ion mode with a cone voltage of 150v.
  3. Deconvolute the raw data using appropriate software. Calculate the difference in mass between the modified and the unmodified antibodies.

### 3. Assay Stability of the Dihydropyridazine Linkage in Trastuzumab(TAMRA)_2_ in Serum

1. Filter the serum using a 0.22 μm filter under sterile conditions. You can add 100 units/mL penicillin, and 100 μg/mL streptomycin.
2. Fill the external wells of a 96-well plate with water. In the central wells, mix in triplicate 90 µL of filtered serum with 10 µL of 1 mg/mL trastuzumab(TAMRA)_2_ in PBS at a final concentration of 0.1 mg/mL. Place the plate in an incubator saturated with humidity at 37 °C and 4-5% CO_2_.
3. Every 24 h throughout 5 days, pipet each well thoroughly to mix, take a 5 µL aliquot, flash-freeze with liquid nitrogen, and store at -80 °C.
4. Once all samples have been collected, thaw the aliquots and analyze using an indirect ELISA as previously reported^12^ with some modifications:
  1. Coat a 96-well plate overnight with 0.25 µg/mL HER2 at 4 °C. All other steps are performed at room temperature.
  2. On the following day, wash 5 times with PBS 0.05% tween (PBS-T) and block with 1% bovine serum albumin for 1 h.
  3. Wash and incubate the samples at 1:10000 dilution in PBS for 2 h.
  4. Wash and incubate an anti-TAMRA antibody raised in mouse at 1:2000 dilution in PBS-T with 0.5% BSA for 1 h.
  5. Wash and incubate an anti-mouse HRP conjugate 1:1000 in PBS-T with 0.5% BSA for 1 h.
  6. Add TMB and allow to react 5-10 min.
  7. Stop reaction with 50 µL H_2_SO_4_ 1 M and measure absorbance at 450 nm. Subtract background measured at 570 nm.
  8. Fit a 4-parameter logistic regression curve to standard dilutions and interpolate measurements from samples. Note: The stability of trastuzumab(MMAE)_2_ could be assessed using the same protocol by changing the assay described in 3.3 for a commercial ELISA kit to measure the concentration of the ADC.

### 4. Assess the Cytotoxicity of the ADC

Note: This protocol is based on previously reported assays^23^,^26^ with some modifications.

1. Thaw SK-BR-3 and MCF-7 cells and allow them to settle in p25 flasks containing complete medium, i.e. DMEM supplemented with 10% heat inactivated fetal bovine serum, 100 units/mL penicillin, and 100 µg/mL streptomycin.
2. Split cells at 80-90% confluence (every 3-4 days) at least 2 times before the assay.
3. Two days prior to the assay, fill the outer wells of a 96-well plate with water. Then lift cells with 0.05% trypsin in 0.5 mM EDTA, centrifuge 3 min at 250 x g, resuspend in new medium and seed 3000 cells in 100 µL per (empty) well in the 96-well plates.
4. Two days after seeding the cells, prepare 10 serial dilutions of all samples in triplicate with 0.1% DMSO in complete medium. Consider the following samples and controls: trastuzumab(MMAE)_2_, trastuzumab(CypK)_2_, trastuzumab wild type, tetrazine-vcMMAE and MMAE.
5. Add 100 µL of each sample in each well and incubate for 5 days at 37 °C and 4-5% CO_2_.
6. On day 5, measure cell viability. To this end, use a commercial kit to lyse the cells and measure the ATP released. Plot the percentage of signal with respect to control cells treated with 0.1% DMSO. Note: ADCs can be incubated for 5 days in serum and assayed for cytotoxicity to prove functional stability. CAUTION: MMAE is highly toxic. Therefore use gloves and goggles when handling MMAE derivatives. If you use unmodified MMAE as a control in your experiment, when you prepare the stock solution in DMSO, handle the solid product inside a fume hood.

## Representative Results

The reported transient expression system (Figure 1a) yields 22 ± 2 mg of trastuzumab(CypK)_2_ per liter of culture medium, which represents 2/3 of the wild type antibody produced under the same conditions (Figure 1c). The stable cell line can increase this yield up to 31 ± 2 mg/L^21^.

Trastuzumab(CypK)_2 _can be conjugated with tetrazine-vcMMAE, which yields quasi homogeneous trastuzumab(MMAE)_2_ within 3 h at 25 °C (Figure 2). The high hydrophobicity of this cytotoxin requires addition of 10% acetonitrile when 5 or more molar equivalents of toxin per CypK are used. Alternatively, the cycloaddition is also completed within 20 h using 2 equivalents of tetrazine-vcMMAE without acetonitrile (Figure 2c). Trastuzumab(CypK)_2_ reacts with tetrazine-TAMRA within 2 h at 25 °C and 3-6 h are required when the temperature is decreased to 4 °C (Figure 3c).

The expected DAR for trastuzumab(MMAE)_2_ measured by HPLC-HIC is 1.9 (Figure 2b). The peak initially observed in the chromatogram at 8.0 min represents the unconjugated antibody (DAR 0) and should have completely disappeared when the reaction is completed. The species with DAR 1 elutes at 9.1-9.6 min and should have an area < 10% after 3 h; and the target product with DAR 2 has a retention time of 10.5-11.0 with an expected area > 90%. The mobility shift and fluorescence in SDS-PAGE gels confirms the incorporation of TAMRA (Figure 3b) and the identity of the immunoconjugates is verified by LC-MS (Figure 2d**-e** and Figure 3d).

Incubation of trastuzumab(TAMRA)_2_ for 5 days in human serum and subsequent analysis by ELISA confirms that the payload remains attached to the antibody (Figure 4b). Regarding the cytotoxicity assay, trastuzumab(MMAE)_2 _shows high potency in SK-BR-3 (HER2 high) breast cancer cells, with a half maximal effective concentration (EC_50_) of 55 ± 10 pM (Figure 4d). Trastuzumab(MMAE)_2_ maintains the cytotoxicity after 5 days of incubation in human serum (Figure 4c). Conversely, when the ADC is assayed on MCF-7 (HER2 low) the EC_50_ is 200-fold lower (Figure 4d). The wild type antibody, trastuzumab(CypK)_2 _and tetrazine-vcMMAE show extremely low toxicity (**Figures 4d and 4e**), whereas MMAE displays high non-selective cytotoxicity in both cell lines (Figure 4e).

**Figure:**
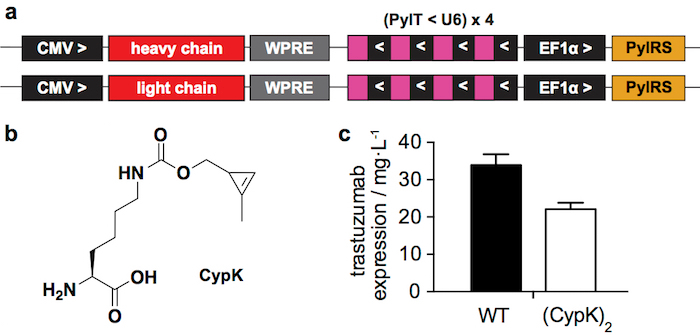

**Figure 1: Transient expression system. A.** Relevant regions of the plasmids used for transient transfection in HEK293 cells. CMV: cytomegalovirus promoter, WPRE: Woodchuck Hepatitis Virus Posttranscriptional Regulatory Element, PylT: pyrrolysyl tRNA, U6: specific promoter, PylRS: pyrrolysil tRNA synthetase, > and <: direction of transcription. **B.** N^ε^-[((2-methylcycloprop-2-en-1-yl)methoxy)carbonyl]-L-lysine (CypK). **C.** Expression yields of wild type (WT) trastuzumab and trastuzumab(CypK)_2_ as measured in a western blot after protein A purification. Error bars represent the standard deviation of biological triplicates. This figure has been modified with permission from Oller-Salvia* et al. *2018^21^. Please click here to view a larger version of this figure.

**Figure:**
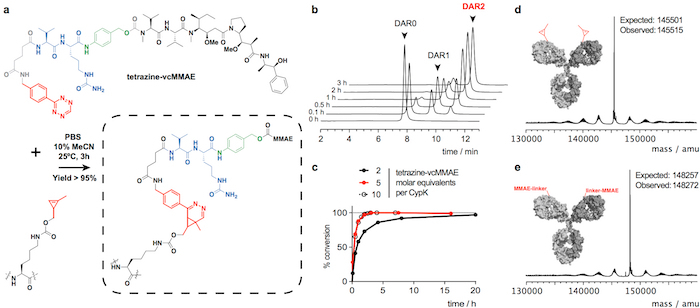

**Figure 2: Conjugation of trastuzumab(CypK)_2_ with tetrazine-vcMMAE. A.** Inverse electron-demand Diels-Alder cycloaddition between the CypK residue in the antibody and the tetrazine derivative of MMAE. The reacting groups are highlighted in red, *p*-aminobenzylalcohol is depicted in green and the valine-citrulline dipeptide in blue. **B.** HPLC-HIC chromatograms showing the progress of the conjugation of antibody. **C.** Degree of conversion with respect to the maximum DAR of 1.9 using different reagent concentrations. **D-E.** Deconvoluted mass spectra of the full-length antibody before and after the conjugation. Trastuzumab(CypK)_2_ was obtained using the stable cell line. This figure has been modified with permission from Oller-Salvia* et al. *2018^21^. Please click here to view a larger version of this figure.

**Figure:**
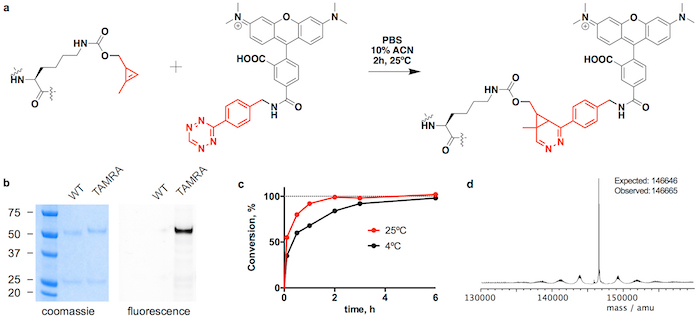

**Figure 3: Conjugation of trastuzumab(CypK)_2_ with tetrazine-TAMRA. A.** Inverse electron-demand Diels-Alder cycloaddition between the CypK residue in the antibody and the tetrazine derivative of TAMRA. **B.** SDS-PAGE gels showing the mobility shift and the in-gel fluorescence originated by the conjugation of TAMRA. **C.** Conjugation kinetics at two different temperatures. **D.** Deconvoluted mass spectrum of trastuzumab(TAMRA)_2. _Trastuzumab(CypK)_2_ was obtained using the stable cell line. This figure has been modified with permission from Oller-Salvia* et al. *2018^21^. Please click here to view a larger version of this figure.

**Figure:**
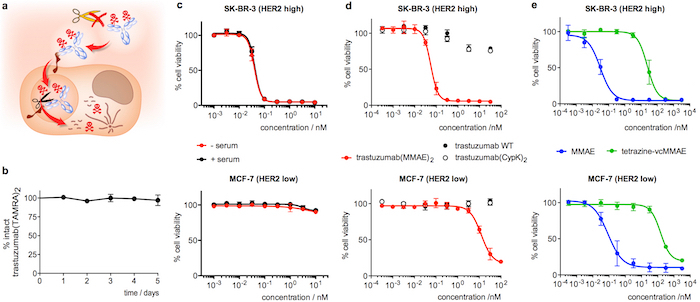

**Figure 4: Stability in serum and cytotoxicity of trastuzumab conjugates. A.** Cartoon highlighting the features desired in an internalizing ADC. **B.** Stability of trastuzumab(TAMRA)_2 _in human serum as measured in ELISA. **C.** Cell viability assay with trastuzumab(MMAE)_2_ after 5 days in human serum (+ serum, black). A control sample incubated in PBS instead of serum (- serum, red) was included in the same assay. **D.** Cell viability assay with freshly diluted antibody samples. **E.** Cell viability assay with freshly diluted MMAE derivatives. Error bars represent the standard error of the mean of 3 independent experiments. This figure has been modified with permission from Oller-Salvia* et al. *2018^21^. Please click here to view a larger version of this figure.

## Discussion

The transient expression procedure to produce trastuzumab(CypK)_2_ described in this protocol is simple and allows for high modularity. The yields obtained are within the ones expected in an academic setting^27^ and stable cell lines can be generated to further boost the production yield^21^. During expression, concentrations of CypK lower than 5 mM may result into lower ncAA incorporation, and higher amounts may affect cell growth and decrease antibody yields. CypK as a free amino acid has low water solubility, thus it should be first dissolved at 100 mM in 0.1 M NaOH and then added to the culture medium. After diluting CypK in the medium and before adding it to cells, it is critical to neutralize the medium with HCl and filter to sterilize. Subsequently, using the transfection reagents specified in this protocol and following the incubation times recommended by the manufacturer is important for a high-yielding expression. For further details on transient expression of human antibodies, the reader is referred to other published protocols^31^,^32^.

When the antibody is purified, a high excess of protein A resin is required as indicated to ensure full antibody pull down from the supernatant. In order to prevent the precipitation of trastuzumab during elution, it is recommendable to use a solution with high buffering capacity, dilute immediately with PBS and exchange the buffer avoiding excessive concentration. Always keep the antibody <5 mg/mL.

The conjugation of trastuzumab(CypK)_2_ with tetrazine-vcMMAE is faster than most reactions reported with other bioorthogonal handles for ADCs. Moreover, this cycladdition occurs under very mild conditions: room temperature or lower and physiological pH. It is important to dilute the DMSO stock solutions of the reactants with acetonitrile prior to addition of PBS and the antibody; otherwise the tetrazine derivatives will precipitate. Acetonitrile is required only due to the high hydrophobicity of MMAE and TAMRA, but less hydrophobic molecules may not need the addition of a co-solvent. Alternatively, tetrazine-vcMMAE can be conjugated without acetonitrile and only 2 molar equivalents of tetrazine-vcMMAE within 20 h. This little amount of toxin could involve a substantial decrease in the manufacturing cost of ADC when compared to current ncAA-based technologies. Trastuzumab(CypK)_2_ is fully reactive for at least 4 months when preserved at 4 °C.

HPLC-HIC enables an accurate determination of DAR since MMAE is highly hydrophobic and provides an excellent resolution of the peaks corresponding to antibody conjugates with 0, 1 and 2 toxins. Unreacted tetrazine-vcMMAE elutes around 13.7 min and is detected at 280 nm. This technique requires a starting material with high purity. Moreover, it is not recommendable to quench the reaction with other tetrazine-reactive molecules such as BCN-OH since they can alter the retention times and the shape of the peaks. It is essential that the salt concentration of the samples matches the one in the mobile phase at the start of the gradient in order to obtain a good separation, especially if more than 10-20 µL are injected.

Regarding the LC-MS analysis, deglycosylation of the antibody samples is required to obtain a single peak upon deconvolution of the raw spectrum. The accuracy of the total antibody and ADC masses may vary depending on the callibration of the instrument. Hence, in order to calculate the mass for the modification, subtract the mass obtained for the unmodified antibodies from the one obtained for the ADC. Modern high-resolution mass spectrometers should provide a relative error below 1:10000. Although LC-MS can also be used to calculate the ratio between the different species, this value is usually an overestimation because the modification may affect the ionization capacity of the species generated and low amounts of impurities may not be detected.

The stability of the linker in ADCs is critical because the premature release of the drug results in higher toxicity and lower efficacy; the free cytotoxin damages healthy tissues and the naked antibody competes with the armed one for the target binding sites on diseased cells. A release below 5%, which is within the variability of the stability assay, should be expected.

Finally, the selectivity of an ADC targeting HER2 such as trastuzumab(MMAE)_2_ can be assessed by comparing the cytotoxicity in SK-BR-3 cells (HER2 high) and MCF-7 cells (HER2 low) since the latter express 15-fold less HER2 receptors than the former^28^. The immunoconjugate is expected to result into a cell viability at least 2 orders of magnitude lower in SK-BR-3 when compared to MCF-7. The EC_50_ in SK-BR-3 should be in the two-digit nanomolar range reflecting the high potency of this ADC^29^,^30^. The unmodified antibody, either trastuzumab(MMAE)_2_ or trastuzumab, should show no toxicity in this assay. Tetrazine-vcMMAE should have an effect 3 orders of magnitude lower than the ADC since the linker removes the activity of the peptidomimetic toxin. Conversely, because MMAE is able to permeate the cell membrane^30^, it should have a similar toxicity to the ADC but display no discrimination between HER2 high and HER2 low cell lines. Moreover, if this assay is performed after a 5-day incubation of the ADC in serum, it can be used to provide a functional proof of the stability of the linker: a release of the toxin would result into either a decrease in ADC efficiency in SK-BR-3 if MMAE was released with part of the linker or a decrease in the selectivity if the linker was cleaved in a traceless fashion.

The ADC technology described herein allows the efficient and site-specific incorporation of a cyclopropene derivative of lysine into IgG1s. Following a facile purification, antibodies can be rapidly conjugated with tetrazine-containing molecules, yielding homogenous products. Due to the small size and high reactivity of the cyclopropene minimal handle, this method should enable the conjugation of sterically hindered payloads. The resulting immunoconjugates are stable in serum and are highly potent and selective. Overall, CypK enables a fast, site-specific and stable bioorthogonal linkage for antibody and other protein conjugates to be used in therapy or diagnosis.

## Disclosures

The authors have nothing to disclose.
